# Anchoring the Self to the Body in Bilateral Vestibular Failure

**DOI:** 10.1371/journal.pone.0170488

**Published:** 2017-01-20

**Authors:** Diane Deroualle, Michel Toupet, Christian van Nechel, Ulla Duquesne, Charlotte Hautefort, Christophe Lopez

**Affiliations:** 1 Aix Marseille Univ, CNRS, LNIA, FR3C, Marseille, France; 2 IRON, Institut de Recherche en Oto-Neurologie, Paris, France; 3 Centre d’Explorations Fonctionnelles Oto-Neurologiques, Paris, France; 4 Unité Troubles de l’Equilibre et Vertiges, CHU Brugmann, Bruxelles, Belgique; 5 Unité de Neuro-Ophtalmologie, CHU Erasme, Bruxelles, Belgique; 6 Clinique des Vertiges, Bruxelles, Belgique; 7 Service ORL, Hôpital Lariboisière, Paris, France; Universite de Bretagne Occidentale, FRANCE

## Abstract

Recent findings suggest that vestibular information plays a significant role in anchoring the self to the body. Out-of-body experiences of neurological origin are frequently associated with vestibular sensations, and galvanic vestibular stimulation in healthy participants anchors the self to the body. Here, we provide the first objective measures of anchoring the self to the body in chronic bilateral vestibular failure (BVF). We compared 23 patients with idiopathic BVF to 23 healthy participants in a series of experiments addressing several aspects of visuo-spatial perspective taking and embodiment. In Experiment 1, participants were involved in a virtual “dot-counting task” from their own perspective or the perspective of a distant avatar, to measure implicit and explicit perspective taking, respectively. In both groups, response times increased similarly when the avatar’s and participant’s viewpoint differed, for both implicit and explicit perspective taking. In Experiment 2, participants named ambiguous letters (such as “b” or “q”) traced on their forehead that could be perceived from an internal or external perspective. The frequency of perceiving ambiguous letters from an internal perspective was similar in both groups. In Experiment 3, participants completed a questionnaire measuring the experienced self/body and self/environment “closeness”. Both groups reported a similar embodied experience. Altogether, our data show that idiopathic BVF does not change implicit and explicit perspective taking nor subjective anchoring of the self to the body. Our negative findings offer insight into the multisensory mechanisms of embodiment. Only acute peripheral vestibular disorders and neurological disorders in vestibular brain areas (characterized by strong multisensory conflicts) may evoke disembodied experiences.

## Introduction

As suggested by Merleau-Ponty’s statement *“I am not in front of my body*, *I am in my body*, *or rather I am my body”* [1], common self experience is characterized by strong anchoring of the self to the body. Pioneer [2] and current [3] psychological studies revealed that adults and children invariably locate their self within their body boundaries. Only in rare clinical conditions as well as during drug use, meditation, and sleep paralysis do individuals claim that their self is located outside their physical body (i.e., out-of-body experience) [4].

Current neuroscientific models of self-location propose that the accurate integration of visual, tactile, proprioceptive, interoceptive, motor and vestibular signals underpins the experience of an embodied self [5]. In support of these models, clinical observations show that abnormal multisensory integration in epileptic, and brain-damaged patients may evoke a loss of unity between the self and the body [6–8]. Moreover, experimentally induced conflicts between vision and touch [9–13], or between vision and motor-proprioceptive signals [14], can modify the anchoring of the self to the body in neurologically normal people. However, despite the importance of the vestibular system in encoding self-motion and orientation in space, its contribution to the sense of self has received much less attention than has vision, touch and proprioception.

The last 10 years has seen a growing amount of evidence from research involving neurological patients and healthy participants suggesting that vestibular signals contribute to anchoring the self to the body (for recent reviews, see Ref. [15–17]). First, neurological patients reporting out-of-body experiences often experience concomitant vestibular illusions, such as sensations of floating and elevation of the self [7,16]. In these patients, damaged areas most frequently overlap with key vestibular regions, including the temporo-parietal junction [11] and posterior insula [8]. Second, patients with peripheral vestibular disorders may report an abnormal self–body relationship, which is reminiscent of depersonalization disorders [18–20]. For example, patients with Menière’s disease may report experiences such as *“I feel like I’m outside of myself*. *I feel like I’m not in myself”*, or *“I am not actually being there or having anything to do with my body”* ([21], p. 531–532). Yet, we lack convincing evidence of full-blown disembodiment related to peripheral vestibular disorders [19,22]. Third, experiments involving healthy participants indicate the possibility of manipulating anchoring of the self to the body by using vestibular stimulation. Ferrè *et al*. [23] showed that low-intensity galvanic vestibular stimulation promoted first-person perspective taking in participants who perceived letters drawn on their forehead. This finding suggests that weak vestibular stimulation may increase the natural tendency of the vestibular system to anchor the self to the body.

If vestibular information plays a significant role in anchoring the self to the body, as suggested by the corpus of data summarized above, how do vestibular-defective patients experience self-location? Anecdotal reports have been collected over the last century [18–20], but we have no objective measures of self-body anchoring in vestibular patients according to well-controlled paradigms from cognitive neuroscience. Here, we tested the contribution of vestibular signals to anchoring the self to the body by comparing the performance of patients with chronic, idiopathic, bilateral vestibular failure (BVF) and healthy controls in three experiments addressing several aspects of embodiment. *Experiment 1* measured implicit and explicit visuo-spatial perspective taking in a virtual-reality–based “dot-counting task” [24–26]. *Experiment 2* measured implicit perspective taking in a non-visual task [23,27] and required naming letters drawn on the participant’s forehead and neck. *Experiment 3* measured the experienced closeness between the self and the body by using pictorial descriptions adapted from the Inclusion of Other in the Self (IOS) scale [28]. The rationale and hypotheses for each experiment are reported in details in the subsequent sections.

### Participants with Idiopathic Bilateral Vestibular Failure

We tested a population of 23 patients with idiopathic bilateral vestibular failure (BVF) in a series of experiments (22 participants in Experiment 1, 23 in Experiment 2, and 22 in Experiment 3). The BVF occurred at a mean of 14 ± 12 years before inclusion in the study. At the time of the tests, all patients were adapted to the vestibular loss, which had moderate functional impact on their daily life, although they reported oscillopsia and imbalance in darkness. The clinical status of these patients and their performance in cognitive, postural and oculomotor tasks are described elsewhere [29–31].

The BVF was established on the basis of standard otoneurological examinations including a bithermal caloric test (irrigation of the left and right auditory canals with water at 44°C and 30°C), the video head impulse test (vHIT) [32], and measurement of vestibulo-ocular responses during a pendular test on a rotating chair. The saccular and utricular functions were evaluated for some patients by recording cervical vestibular-evoked myogenic potentials (cVEMPs) over the sternocleidomastoid muscles [33] and ocular vestibular-evoked myogenic potentials (oVEMPs) over the inferior oblique muscles [34], respectively.

All patients had weak responses to the caloric test [mean slow phase eye velocity <5°/s [35]; left ear (mean ± SD): 2.42 ± 2.73°/s, right ear: 2.36 ± 2.53°/s] and reduced responses to the vHIT [mean gain <0.7 [36]; horizontal canals: 0.38 ± 0.19; anterior canals: 0.34 ± 0.17; posterior canals: 0.34 ± 0.15]. Responses to the pendular test were also reduced [mean slow phase eye peak velocity <20°/s; left rotation: 5.89 ± 7.37°/s; right rotation: 4.84 ± 5.11°/s]. Cervical VEMPs were present in the left ear for 9 patients (mean p13-n23 amplitude ± SD: 33.59 ± 42.14 μV) and in the right ear for 12 patients (41.76 ± 44.09 μV). Ocular VEMPs were present in the left ear for 5 patients (0.68 ± 1.34 μV) and in the right ear for 6 patients (0.97 ± 1.61 μV). In conclusion, all patients presented severe bilateral vestibular hypofunction that was not associated with neurological disorders.

### Ethics statement

All participants were informed about the study and gave their written informed consent. Experimental procedures were approved by the local Ethics Committee (Comité de Protection des Personnes Sud-Méditerranée II) and followed the ethical recommendations laid down in the Declaration of Helsinki.

## Experiment 1

Experiment 1 aimed at measuring the degree of anchoring the self to the body by visuo-spatial perspective taking tasks. Recent research has suggested that implicit third-person perspective taking can be evaluated by asking participants to perform visuo-spatial judgments from their own perspective while another, task-irrelevant, person is in their visual environment. Participants spontaneously adopt the viewpoint of the person in their environment. For example, participants instructed to describe the relative position of two objects more often located these objects according to the perspective of a person sitting in front of them [37–39]. This effect was further increased when the other person gazed or acted toward one of the objects.

Here, we compared implicit and explicit visuo-spatial perspective taking tasks in BVF patients and controls by using a virtual “dot-counting task” developed by Samson and colleagues and replicated by others [24–26,40–44]. In this task, participants reported whether the number of dots presented on the walls of a 3D virtual room matched a digit presented in a previous instruction. The environment involved a task-irrelevant avatar. Under conditions where the avatar could “see” a number of dots incongruent with the number of dots visible from the participants’ viewpoint, response times and error rates increased. Several studies confirmed that such effects were due to “altercentric intrusion” [24–26,40,41,43,44], that is, an implicit and unconscious simulation of the avatar’s viewpoint. An opposite effect was reported when participants were explicitly asked to simulate the avatar’s perspective (i.e., to imagine how many dots the avatars would see). Participants were slower and made more errors when the number of balls seen from the avatar’s and participant’s viewpoints was incongruent. This effect, referred to as “egocentric intrusion”, indicates that participants cannot totally ignore their own visuo-spatial perspective.

If vestibular signals are important to anchor the self to the body [23], BVF patients may more easily separate from their own perspective. Accordingly, they may be more prone to implicitly take the avatar’s disembodied perspective (i.e., more altercentric intrusion) and less anchored to their body when required to explicitly take the avatar’s perspective (i.e., less egocentric intrusion). An opposite hypothesis would be that BVF patients would be less likely to implicitly and explicitly adopt the avatar’s perspective because vestibular signals are required for computing a third-person perspective [45]. Preliminary findings were presented in a conference abstract [46].

### Methods

#### Participants

Twenty-two patients with idiopathic BVF participated in the experiment (9 females and 13 males, mean age ± SD: 60 ± 11 years, mean duration of education after high school: 4 ± 2 years). All patients but one were right-handed, as confirmed by the Edinburgh Handedness inventory (mean laterality quotient: 91 ± 30%) [47]. They had normal or corrected-to-normal vision. BVF patients were compared to 22 healthy volunteers matched on age, sex and education level (9 females and 13 males, age: 58 ± 12 years, education: 5 ± 3 years). Healthy participants were all right-handed, (laterality quotient: 94 ± 13%), had normal or corrected-to-normal vision, and no history of vestibular, neurological, or psychiatric disease.

#### Implicit perspective taking task (IPT task)

**Fig 1:**
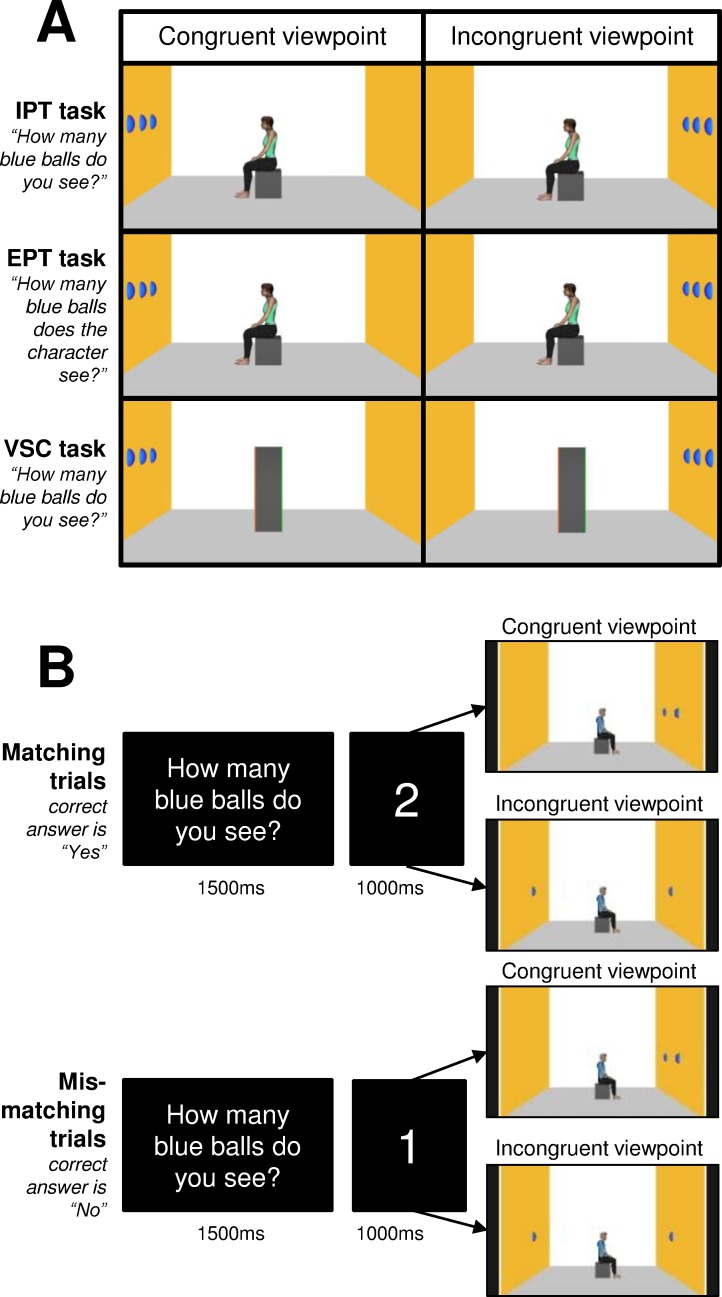
Methods for visuo-spatial perspective-taking tasks (Experiment 1). (A) Examples of visual stimuli used for the tasks of implicit perspective taking (IPT), explicit perspective taking (EPT) task, and visuo-spatial control (VSC) task. Visual stimuli presented a congruent or an incongruent viewpoint of the avatar with the participant’s viewpoint. (B) Participants indicated whether the number of balls seen from their viewpoint (IPT and VSC tasks) matched (i.e., matching trials) or did not match (i.e., mismatching trials) the number presented in the instruction.

Visual stimuli consisted of a colored 3D rendering of a room with three visible walls. The left and right walls were yellow and contained from 0 to 3 blue balls aligned horizontally. In the middle of the room and at the center of the screen, an avatar was shown sitting on a cube placed on the room floor. Two sets of pictures were created: female avatars were always shown to female participants, and male avatars were always shown to male participants. The avatar faced the left or right wall of the 3D room. The spatial arrangement of the balls was manipulated to create situations where the participant and avatar could “see” the same number of balls on the walls (i.e., congruent viewpoint), or a different number of balls (i.e., incongruent viewpoint) (**Fig 1**). In total, for both female and male avatars and for both avatar orientations (i.e., facing the left or right wall), 10 visual stimuli were created to balance the number of trials with congruent and incongruent viewpoints (following procedures from Ref. [24]).

Visual presentation was controlled, and responses were collected by using PsychoPy2 v1.82.01 [48]. Each trial started with the presentation of a white fixation cross on a black background for 750 ms. This was followed by the presentation of the question *“How many blue balls do you see*?*”* for 1500 ms and the presentation of a number (0, 1, 2 or 3) for 1000 ms. Then, one of the visual scenes was presented. Participants were instructed to indicate as quickly and accurately as possible whether the number of balls they saw matched the number specified after the question. The response time was not limited. Participants pushed one of two buttons on a keyboard to respond: half of the participants had to press a button with their right index finger to answer “yes” or another button with their right middle index finger to answer “no”; the other participants had a reverse configuration for the response buttons. As soon as participants pressed a button, the visual scene disappeared and the next trial started. Although participants had to count the number of balls according to their first-person perspective, the presence of the avatar in the visual scene allowed for measuring implicit third-person perspective taking (IPT), i.e. the extent to which the avatar’s viewpoint interfered with the participant’s viewpoint.

In half of the trials (“matching trials”), the number specified after the question matched the number of balls visible from the participant’s viewpoint (**Fig 1B**). For the trials involving a congruent viewpoint, the number shown after the question corresponded to the quantity of balls visible from both the participant’s and avatar’s viewpoints. For the trials involving an incongruent viewpoint, the number corresponded to the quantity of balls visible only from participant’s viewpoint. In the other half of the trials (“mismatching trials”), the number specified after the question differed from the quantity of balls the participant could see. For the trials involving a congruent viewpoint, the number shown after the question corresponded to one of the three quantities of balls that did not match the quantity of balls visible from the participant’s and avatar’s viewpoints. For the trials involving an incongruent viewpoint, the number corresponded to the quantity of balls visible only from the avatar’s viewpoint. Following the procedures from Ref. [24], we created six “filler trials” corresponding to a visual scene containing no ball on the left and right walls and for which the number “0” shown after the question was the correct answer. Visual stimuli were presented as 35 × 20 cm images on a computer screen.

#### Explicit perspective taking task (EPT task)

Visual stimuli were identical to the 10 stimuli created for the IPT task, with the same avatar at the center of the screen facing one of the walls (**Fig 1A**). Here, the instruction differed: participants were explicitly asked to take the avatar’s viewpoint (explicit third-person perspective taking, EPT).

Each trial started with the presentation of a white fixation cross on a black background for 750 ms. This was followed by the presentation of the question *“How many blue balls does the character see*?*”* for 1500 ms and the presentation of a number (0, 1, 2 or 3) for 1000 ms. Then, one of the visual scenes was presented. Participants were instructed to indicate as quickly and accurately as possible whether the number of balls seen by the character matched the number specified after the question. Participants responded using the same two buttons on a keyboard as for the IPT task.

As for the IPT task, we included trials in which the participant and the avatar could “see” the same number of balls (i.e., congruent viewpoint) or a different number of balls (i.e., incongruent viewpoint). Half of the trials were “matching trials” and the other half were “mismatching trials” and we included six filler trials.

#### Visuo-spatial control task (VSC task)

To control for visuo-spatial and attentional bias in the IPT and EPT tasks, participants completed a visuo-spatial control task (VSC task) involving neither implicit nor explicit perspective taking. Here, a grey rectangle (a geometric shape devoid of social meaning) replaced the avatar at the center of the screen (for similar procedures, see Ref. [24,25,49]) (**Fig 1A**). The control task aimed to control for (1) differences in visual processing, motor response accuracy and speed between BVF patients and controls and (2) visuo-spatial effects that may account for longer response times in incongruent trials (balls on one wall or on two opposite walls) as compared to congruent trials (balls always on the same wall).

An arbitrary “orientation” of the rectangle in the room was created by coloring the left and right sides of the rectangle in orange or green. Half of the participants were presented with the right side of the rectangle in orange and the left side in green, and other participants were presented with the opposite orientation. Spatial arrangements of the balls labeled “congruent viewpoint” in the IPT task were considered the congruent viewpoint in the control task and vice versa for trials labeled “incongruent viewpoint” in the IPT task (**Fig 1A**).

As for the IPT task, the VSC task involved matching trials in which the number specified after the question matched the number of balls visible from the participant’s viewpoint. In the other half of the trials (i.e., mismatching trials), the number specified after the question differed from the quantity of balls the participant could see. Six filler trials were also presented. The VSC task involved the same instructions, experimental procedures and timing of events presentation as for the IPT task.

#### Experimental procedures

For all three tasks, participants sat on a chair facing a screen placed on a table. Their heads were aligned with the center of the screen, which was at a viewing distance of 70 cm. A keyboard was placed on the table in front of participants.

For each task, participants completed a total of 78 trials presented in random order in 2 blocks of 39 trials. There was an equal number of matching trials (*n* = 39) and mismatching trials (*n* = 39), and an equal number of trials with congruent (*n* = 36) and incongruent (*n* = 36) viewpoints. Before the experiment, participants completed a training session consisting of a random selection of 20 trials for familiarization with the keyboard and experimental procedures.

Participants first performed the VSC task then the IPT task and the EPT task. This order was chosen because our pilot experiments [50] and other studies [25,49] showed that performing the IPT or EPT tasks first changes reaction times in a control task presenting a non-corporeal object (i.e., a rectangle or an arrow). Accordingly, since the control task was a baseline to measure the participant’s ability to process space, it was always conducted first. To allow for between-group comparisons, the sequence was identical for the BVF patients and healthy controls.

#### Data analysis

We calculated the mean response time and percentage of correct answers for the matching trials. Data for mismatching trials and filler trials were discarded from the analysis according to previous studies [24]. Trials yielding incorrect answers were discarded from the analysis of the response times and we removed trials for which response times exceeded 2 standard deviations of the participant’s grand average. We focused on response times, shown to be more sensitive than accuracy to multisensory conflicts [51–53]. For the three tasks, response times were analyzed by repeated-measures ANOVAs with Statistica, Version12 SP3 (StatSoft Inc.), with Viewpoint (congruent vs incongruent) as a within-subject factor and with Group (BVF patients vs controls) and Gender (female vs male) as between-subject factors. Main effects and interactions were considered significant at p<0.05. We also calculated a congruency effect (CE), adapted from the cross-modal CE used to investigate visual-tactile and visuo-vestibular conflicts [51–53]. For each of the three tasks, CE was calculated as the difference in response times between the incongruent and congruent viewpoints.

### Results

#### IPT task

**Fig 2:**
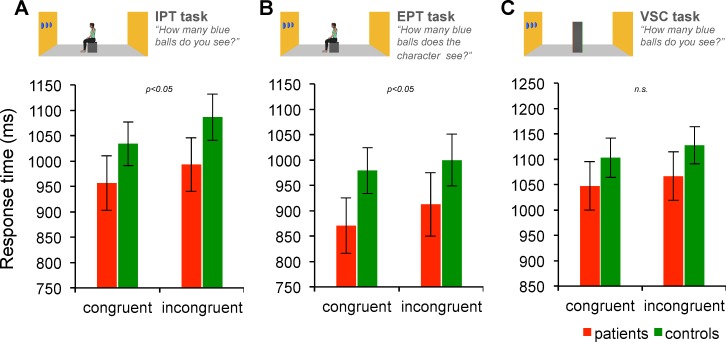
Results for the visuo-spatial perspective-taking tasks (Experiment 1; Response times). Histograms represent the effect of the within-subject factor Viewpoint, which was significant for the implicit perspective-taking (IPT) task (p<0.05) and the explicit perspective-taking (EPT) task (p<0.05), but not for the visuo-spatial control (VSC) task (n.s.: not significant). Data for patients and controls are shown separately for illustration purposes only. Vertical bars represent the standard error of the mean.

Results showed a main effect of Viewpoint (F_1,40_ = 22.87, p<0.0001, η^2^_p_ = 0.36). As predicted, the mean response time was significantly longer when participant’s and avatar’s viewpoints were incongruent (mean ± SD: 1040 ± 234 ms) than congruent (995 ± 230 ms), thereby showing a typical pattern of “altercentric intrusion” (**Fig 2A**). There was no main effect of Group (F_1,40_ = 1.27, p = 0.27, η^2^_p_ = 0.03) and no Viewpoint × Group interaction (F_1,40_ = 0.90, p = 0.35, η^2^_p_ = 0.02), showing no effect of vestibular deficits on altercentric intrusion. There was no main effect of Gender (F_1,40_ = 1.38, p = 0.25, η^2^_p_ = 0.03), but a significant Viewpoint × Gender interaction (F_1,40_ = 4.43, p<0.05, η^2^_p_ = 0.10). Although response times were longer with incongruent than congruent trials for both females (planned comparison: F_1,40_ = 20.07, p<0.0001) and males (F_1,40_ = 4.38, p<0.05), the statistical difference was stronger in females. In addition, the CE was numerically larger for females (70 ± 63 ms) than males (27 ± 67 ms).

#### EPT task

As predicted, again we found a main effect of Viewpoint (F_1,40_ = 10.61, p<0.01, η^2^_p_ = 0.21), with significantly longer response times when the participant’s and avatar’s viewpoints were incongruent (mean ± SD: 956 ± 268 ms) than congruent (925 ± 239 ms). This finding indicates a typical pattern of “egocentric intrusion” (**Fig 2B**). We found no main effect of Group (F_1,40_ = 1.18, p = 0.28, η^2^_p_ = 0.03) and no Viewpoint × Group interaction (F_1,40_ = 0.50, p = 0.49, η^2^_p_ = 0.01), which again shows no effect of vestibular deficits on altercentric intrusion, and no effect of Gender (F_1,40_ = 0.44, p = 0.51, η^2^_p_ = 0.01).

#### VSC task

In contrast to IPT and EPT tasks, analysis of the response times for the *VSC task* depicting a non-human object revealed no effect of Viewpoint (F_1,40_ = 2.53, p = 0.12, η^2^_p_ = 0.06). Thus, response times did not differ for incongruent (1097 ± 200 ms) and congruent (1075 ± 203 ms) viewpoints (**Fig 2C**). We found no significant effect of Group (F_1,40_ = 0.66, p = 0.42, η^2^_p_ = 0.02), no Viewpoint × Group interaction (F_1,40_ = 0.08, p = 0.77, η^2^_p_<0.01) and no effect of Gender (F_1,40_ = 0.52, p = 0.47, η^2^_p_ = 0.01).

#### Congruency effects

**Fig 3:**
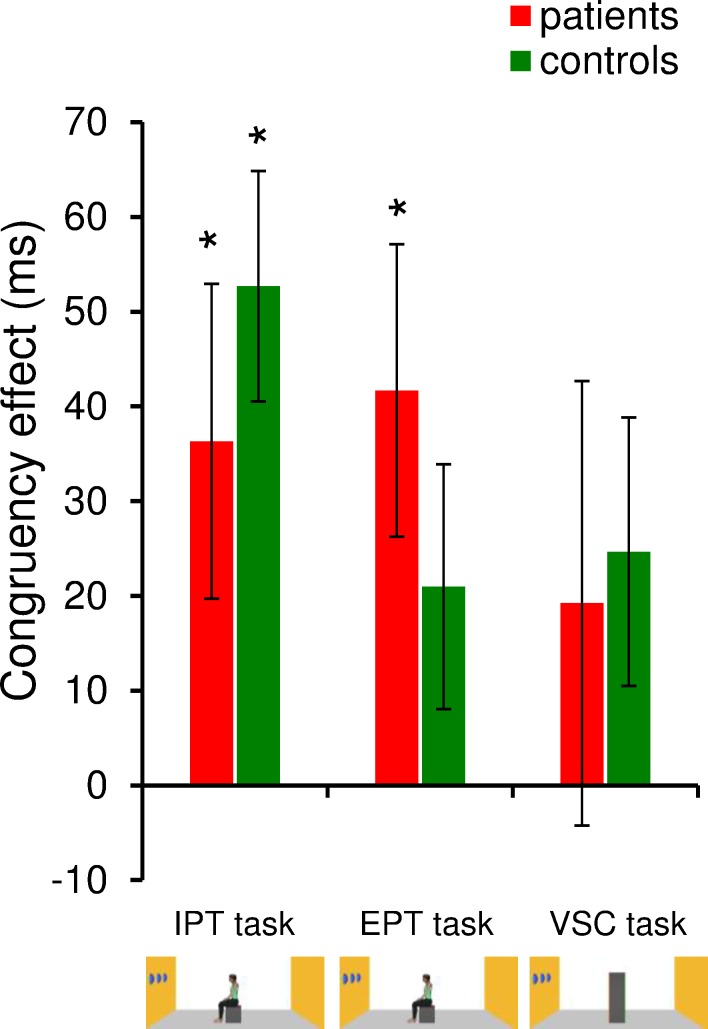
Results for the visuo-spatial perspective-taking tasks (Experiment 1; Congruency effects). Histograms represent the average congruency effect (incongruent viewpoint minus congruent viewpoint) calculated for the implicit perspective-taking (IPT) task, explicit perspective-taking (EPT) task, and visuo-spatial control (VSC) task for patients and controls. * indicates significant differences with respect to zero (t-test). Vertical bars represent the standard error of the mean.

We compared the CE between groups for both perspective taking tasks and VSC tasks (**Fig 3**). Although the CE for the IPT task was numerically lower for the BVF patients (37± 78 ms) than controls (53 ± 57 ms), which suggests reduced altercentric intrusion for patients, the difference was not statistically significant (F_1,42_ = 0.63, p = 0.43, η^2^_p_ = 0.02). An opposite trend was found for the EPT task, with numerically higher CE for patients (42 ± 72 ms) than controls (21 ± 61 ms), which suggests increased egocentric intrusion for patients, but the difference was not statistically significant (F_1,42_ = 1.06, p = 0.31, η^2^_p_ = 0.01). Post-hoc analyses revealed that CEs were significantly different from zero for the perspective taking tasks (except for controls in the EPT task) but never for the VSC task.

## Experiment 2

Experiment 2 was designed to measure implicit perspective taking in BVF patients using a tactile task instead of a visuo-spatial task, as in Experiment 1 and in previous studies [54–56]. We adapted a tactile perception task referred to in the literature as a “graphaesthesia” task. The task consists of drawing ambiguous letters (such as d, b, p and q) on the participant’s forehead directly with the experimenter’s finger [57], a cotton bud [23], or a mechanical device [58]. Participants may perceive letters drawn on their forehead from an egocentric, first-person perspective (e.g., they perceive the letter “d” after the letter “b” is drawn on their forehead) or from a disembodied, third-person perspective (e.g., they perceive the letter “d” after the letter “d” is drawn) (reviewed in [59]). An early study by Natsoulas and Dubanoski [27] revealed that 70% of participants experienced ambiguous letters drawn on their forehead according to a first-person perspective. Interestingly, this proportion changed depending on the site of stimulation and the spatial orientation of stimulated body parts [27,60–62]. For example, only 13% of participants used a first-person perspective when letters were drawn on the back of their head, whereas about 50% of participants used a first-person perspective for letters drawn on the side of their head [27]. Altogether, these data indicate that interpreting tactile patterns on the skin varies across participants and may reflect sensory and cognitive styles, such as those involved in visual field dependence/independence. Accordingly, the graphaesthesia task constitutes a valid measure of implicit perspective taking [23,60].

Two opposite predictions can be made regarding the consequences of BVF in the graphaesthesia task: (1) If vestibular signals are involved in simulating another person’s perspective, as suggested by healthy participant research [45], the lack of vestibular information in BVF patients may promote tactile perception according to a first-person perspective. (2) Conversely, if vestibular signals anchor the self to the body, as suggested by the effect of galvanic vestibular stimulation in healthy participants [23], BVF patients without vestibular signals may more easily take a disembodied viewpoint.

### Methods

#### Participants

Twenty-three BVF patients (9 females and 14 males, mean age ± SD: 61 ± 11 years, 22 right-handed and 1 left-handed, Edinburgh Handedness inventory [47]: 90 ± 30%, duration of education: 4 ± 2 years) and 23 healthy volunteers (mean age: 59 ± 12 years, all right-handed, Edinburgh Handedness inventory: 93 ± 15%, duration of education: 6 ± 3 years) participated.

#### Tactile stimuli and experimental procedures

**Fig 4:**
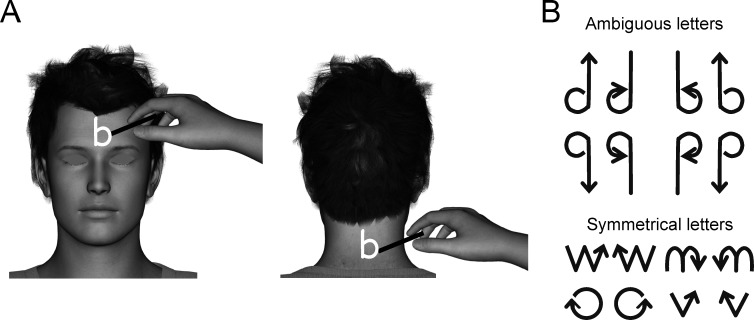
Methods for the graphaesthesia task (Experiment 2). **(A)** The experimenter drew letters on the participants’ forehead and neck by using a cotton bud while participants kept their eyes closed. **(B)** Letters included ambiguous, non-symmetrical letters (b, d, p, q) and non-ambiguous, symmetrical letters (w, n, o, v) that were all drawn in the canonical direction of writing or in the reverse direction, starting from the opposite end of the letter. All letters were drawn by using a single and continuous hand motion.

Procedures for this graphaesthesia task were adapted from those used by Ferrè *et al*. [23] and by Natsoulas and Dubanoski [27]. Before the experiment, participants were verbally instructed that the experimenter would draw letters on their forehead or their neck (on the back of the head below the hairline) by using a cotton bud (**Fig 4A**). Participants were informed that only one of those letters–d, b, p, q, n, v, w and o–would be drawn on their skin, and they were instructed to report as spontaneously as possible the letter they experienced (**Fig 4B**). The experimenter traced the letters by using a cotton bud in a single continuous motion on the skin. To increase the difficulty of the task and to not cue participants to select a strategy based on the direction of the writing, all letters were drawn with the motion starting from one or the other end of each letter, so that letters were traced according to a canonical or non-canonical direction of writing. Thus, participants first had to create a representation of the global shape of the letter before giving an answer, because the direction of the writing was uninformative.

Participants were comfortably seated on a chair and were instructed to close their eyes throughout the recording session. In one session, the experimenter sat in front of the participant and drew the letters on the central part of the participant’s forehead. In the other session, the experimenter sat at the back of the participant and drew the letters on the participant’s neck. Each session comprised 48 trials, including 32 presentations of ambiguous letters (8 presentations of d, b, p and q) and 16 presentations of non-ambiguous letters (4 presentations of n, v, w and o). Letters with their direction of drawing were presented on a computer screen to the experimenter in a randomized order by using PsychoPy2 (v1.82.01) [48]. The experimenter used a keyboard to manually enter participants’ verbal responses, which were saved by using PsychoPy and processed offline.

#### Data analysis

To measure the degree of anchoring the self to the body, we calculated the proportion of ambiguous letters (d, b, p and q) that were experienced from a first-person perspective (e.g., when participants reported the letter “q” after the experimenter drew the letter “p” on their skin) [23]. The ability to correctly represent the pattern of letters drawn on the skin was calculated as the proportion of correct identification of symmetrical letters (n, v, w and o). Data were analyzed by using repeated-measures ANOVAs with the Site of stimulation (forehead vs neck) as a within-subject factor and with the Group (BVF patients vs controls) and Gender (female vs male) as between-subject factors.

### Results

**Fig 5:**
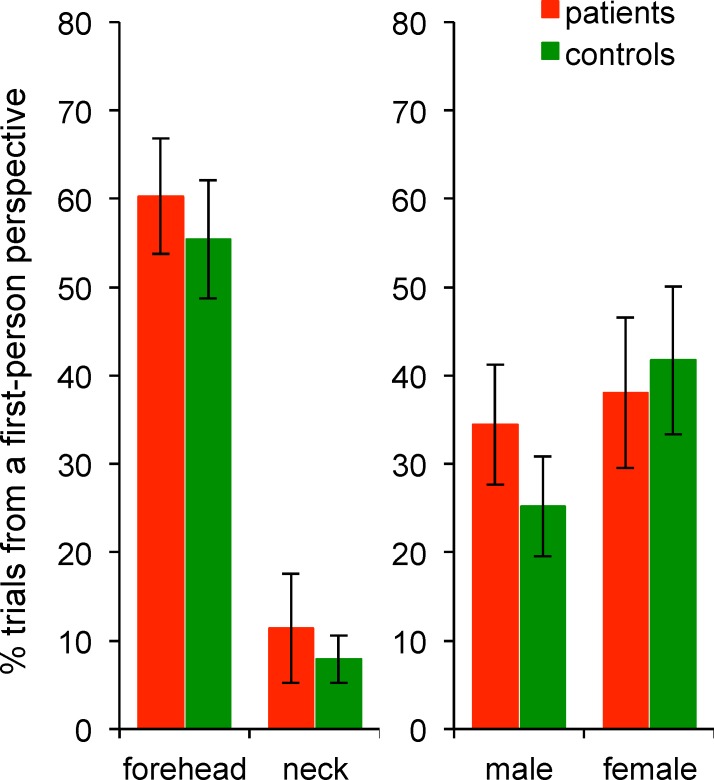
Results from the graphaesthesia task (Experiment 2). Histograms on the left represent the main effect of the within-subject factor Site of stimulation and histograms on the right represent the main effect of the between-subject factor Group. Data from patients and controls are shown separately for illustration purposes only. Histograms represent the mean of the percentage of trials perceived from a first-person perspective and vertical bars represent the standard error of the mean.

Analysis of the proportion of ambiguous letters experienced from a first-person perspective revealed a significant main effect of the Site of stimulation (F_1,42_ = 68.96, p<0.001, η^2^_p_ = 0.62). **Fig 5** shows that letters were more often experienced from a first-person perspective when drawn on the forehead (mean percentage of trials ± SD: 60 ± 31%) and almost never from a first-person perspective when drawn on the neck (10 ± 18%). We found no main effect of Group (F_1,42_ = 0.40, p = 0.53, η^2^_p_ = 0.01) and no significant Site of stimulation × Group interaction (F_1,42_ = 0.01, p = 0.93, η^2^_p_<0.001). There was a significant main effect of Gender (**Fig 5**): letters were more often experienced from a first-person perspective for females (40 ± 15%) than males (30 ± 15%; F_1,42_ = 5.20, p<0.05, η^2^_p_ = 0.11).

Analysis of the proportion of correct identification of symmetrical letters revealed a similar performance for both Groups (F_1,42_ = 0.01, p = 0.94, η^2^_p_ = 0.001) and Genders (F_1,42_ = 0.1, p = 0.75, η^2^_p_ = 0.002). Participants discriminated letters better when drawn on their forehead than on their neck as revealed by a main effect of Site of stimulation (F_1,42_ = 7.62, p<0.01, η^2^_p_ = 0.15). There was no significant interaction.

## Experiment 3

Anecdotal reports in the clinical literature suggest that acute vestibular disorders may impair bodily self-consciousness, for example, evoking sensations that the body feels enlarged, strange, or unreal [18,19]. The subjective content of these symptoms is evocative of depersonalization disorders [21]. Studies involving the depersonalization-derealization questionnaire from Cox and Swinson [63] reported a higher incidence of depersonalization in patients with vestibular disorders than healthy participants and greater incidence of depersonalization in bilateral than unilateral vestibular disorders [64–67]. The Cox and Swinson questionnaire [63] includes items tapping self/body and self/environment relationships, such as *“feeling detached or separated from [the] body”* and *“feeling of detachment or separation from surroundings”* that vestibular patients report significantly more often [64]. Yet, it is notable that there is only few descriptions of complete out-of-body experiences in vestibular disorders [18,19,21,68]. Here, we measured the experienced self/body and self/environment “closeness” in idiopathic BVF patients by using questionnaires.

### Methods

#### Participants

Twenty-two BVF patients (8 females and 14 males, mean age ± SD: 61 ± 11 years, 21 right-handed and 1 left-handed, Edinburgh Handedness inventory [47]: 90 ± 30%, duration of education: 4 ± 2 years) and 22 healthy volunteers (mean age ± SD: 59 ± 12 years, 22 right-handed: 93 ± 13%, duration of education: 6 ± 3 years), who also took part in Experiment 2 filled out a questionnaire.

#### Subjective reports

**Fig 6:**
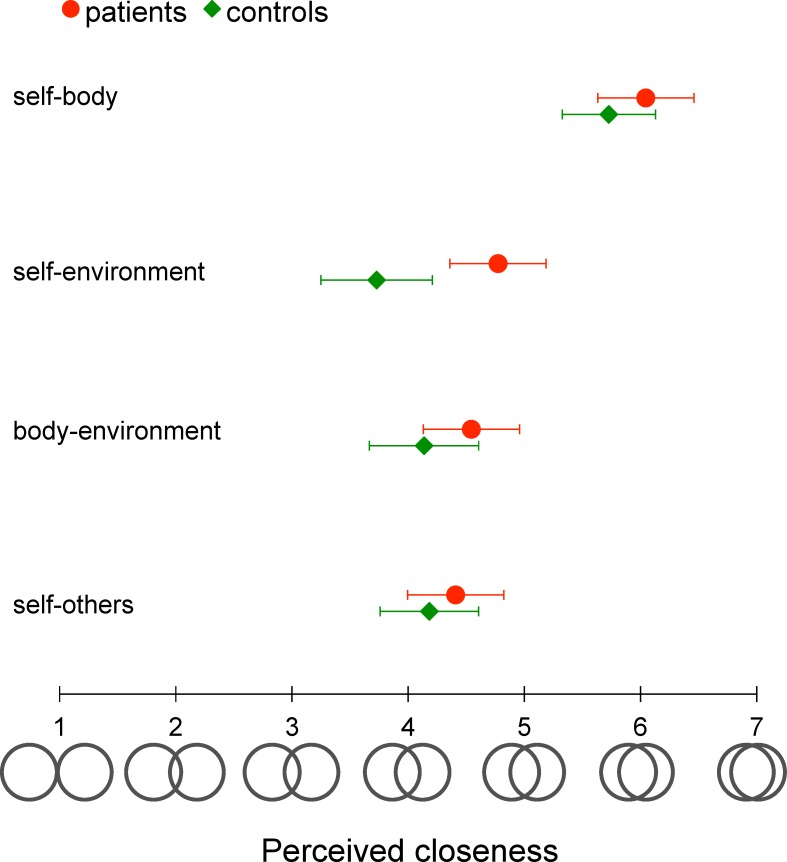
Subjective measures of self-body anchoring (Experiment 3). Pictorial descriptions used to measure the closeness between the self, body, environment and others. Seven pairs of circles shown at the bottom of the figure were presented to participants, who had to indicate which one better represented the perceived degree of “closeness” between two items, such as the self and body. Colored symbols represent the mean of self-reports from patients and controls and horizontal bars represent the standard error of the mean.

Participants completed a four-item questionnaire about the perceived closeness between their self and body (Item 1), self and immediate body environment (Item 2), body and immediate body environment (Item 3), and self and others (Item 4). They were asked to answer in terms of their average perception over the last year. Participants had to select one of seven pictorial descriptions (**Fig 6**) of the degree of closeness between their self and body, for example, whereby two distinct circles represent clear separation between the self and the body (score = 1) and two overlapping circles represent high closeness between their self and body (score = 7). This simple, pictorial description of closeness was adapted from the “Inclusion of Other in the Self” (IOS) scale developed by Aron *et al*. [28] to measure interpersonal closeness.

### Results

For each questionnaire item, the degree of closeness reported was converted into a score ranging from 1 to 7 (**Fig 6**). Scores for the BVF patients and controls were compared by a nonparametric statistical procedure based on the Mann-Whitney U test (i.e., nonparametric alternative to the *t*-test for independent samples). Patients and controls reported a similar degree of closeness between their self and body (U = 235, Z = −0.16, p = 0.87), self and immediate body environment (U = 170, Z = 1.69, p = 0.09), body and immediate body environment (U = 217, Z = 0.59, p = 0.56), and self and others (U = 226, Z = 0.36, p = 0.72). Finally, a separate analysis revealed no modulation of the scores by Gender as a between-subject factor (all U>183, Z<0.99 and p>0.32).

## Discussion

Three experiments revealed that severe bilateral vestibular hypofunction does not change the anchoring of the self to the body. Although negative findings are difficult to interpret, they should be reported more systematically in behavioral and clinical neuroscience [69,70]. Hereafter we discuss our results with respect to current multisensory models of embodiment and compare results from each experiment with earlier studies, while pointing out the limitations of the present study.

### Multisensory mechanisms of embodiment

The negative findings from this study shed light on the multisensory mechanisms of embodiment. Current neuroscientific models of embodiment propose that the common experience of an embodied self relies on normal integration of sensory signals, including vestibular signals [5,16]. These models also predict that a multisensory conflict can evoke the experience that the self is disconnected from the body [5,22].

BVF patients were tested when most of their functional deficits were moderated and they usually did not complain about vertigo and dizziness. Because vestibular information is missing in these patients, it does not contradict nor confirm visual and somatosensory signals during body motions. Accordingly, there should be no sensory mismatch and perceptual incoherence due to bilateral vestibular failure. By contrast, in patients with acute peripheral vestibular disorders, the central nervous system receives signals from the inner ear about self-motion and self-orientation that are incongruent with visual and somatosensory signals, thereby creating sensory mismatch and perceptual incoherence. We propose that abnormal forms of anchoring the self to the body may arise from perceptual incoherence in acute vestibular disorders but not from long-lasting vestibular deafferentation. Indeed, disorders of the bodily self have been reported in clinical conditions such as Menière’s disease [21], recurrent vertigo attacks [68] and epileptic vertigo [71], which are characterized by episodes of strong perceptual incoherence. By contrast, we found no objective measure in the clinical literature showing that bilateral vestibular loss may evoke strong disembodied self-location.

The normal embodiment we found in BVF patients also suggests that the mechanisms underpinning the experience of an embodied self and first-person perspective are robust. Neurologically normal individuals rarely spontaneously report disembodied experiences, unless they experience multisensory conflicts. For example, Pfeiffer *et al*. [12] used visuo-tactile conflicts in healthy participants and could manipulate the direction of their first-person perspectives. Yet, the origin of the first-person perspective invariably remained bound to self-location. In addition, low-intensity galvanic vestibular stimulation promoted a first-person perspective in healthy participants during the graphaesthesia task [23]. This suggests that weak vestibular stimulation may increase the natural tendency of the vestibular system to anchoring the self to the body. In conclusion, we propose that when vestibular information does not conflict with visual and somatosensory signals, as in healthy participants and BVF patients, visuo-spatial processing from a first-person perspective may be unaffected.

We cannot exclude that our negative findings are due to some patients having a severe bilateral vestibular hypofunction rather than a total bilateral vestibular loss. If vestibular signals anchor the self to the body, even a weak residual vestibular function may be enough to maintain a coherent experience of an embodied self. Yet, additional analyses (not presented here) revealed that patients with and without cervical VEMPs had similar performances in the three experiments. Finally, because vestibular signals have been involved in both anchoring the self to the body (egocentric viewpoint) [23] and in simulating another person’s viewpoint (allocentric viewpoint) [45], an alternative explanation for our negative findings could be that these effects tend to cancel each other out. It is unknown from the literature whether vestibular signals are more important for anchoring the self to the body or changing the viewpoint.

The negative findings we report here may also be due to the nature of the task. In Experiments 1 and 2, we used implicit perspective taking tasks. Participants did not explicitly evaluate their self-location and self-identification with an avatar in their environment, as done in experiments using visuo-tactile stimulation [9–14,72,73]. In these experiments, participants received a tactile stimulation on their back or chest while they observed in a head-mounted display an avatar being stroked at the same time on the same body region [9–11,13]. Participants self-identified with the avatar and localized themselves closer to it (i.e., disembodied self-location; for reviews see Ref. [5,74]). Variants of these illusions evoked sensations of body translation, lightness and levitation [11–13], which are reminiscent of otolithic vestibular sensations. By contrast, when tested with variants of the illusions that do not alter self-location, participants do not report vestibular sensations [72,73]. These data suggest a relation between disembodied self-location and vestibular information processing. It is likely that if BVF patients (or patients with unilateral vestibular disorders) were tested using paradigms of visuo-tactile stimulation, their self-location and self-identification would differ from that of healthy controls as they strongly rely on visual information for self-orientation [75]. This hypothesis seems supported by a recent case study by Kaliuzhna *et al*. [68]. A patient with a unilateral vestibular disorder, who already had out-of-body experiences, reported during synchronous visuo-tactile stimulation a stronger sensation that he was floating in the air than control participants. The anchoring of the self to the body should now be investigated in large samples of BVF patients and patients with unilateral vestibular disorders using experimental inductions of out-of-body—like experiences, in order to fully understand the vestibular contributions to embodiment.

### Comparison with previous findings

#### Implicit visuo-spatial perspective taking

As predicted, our data revealed a typical pattern of altercentric intrusion: participants spontaneously adopted the perspective of the avatar to the detriment of visuo-spatial processing from their own perspective (i.e., longer reaction times for incongruent viewpoint). The data also revealed an egocentric intrusion effect, whereby participants did not ignore their own perspective when required to simulate the viewpoint of a distant avatar [24–26,42]. Finally, our data indicate that altercentric and egocentric intrusion effects exist in participants older (mean age 66 years old) than previously tested healthy populations (e.g., mean age was 21 in Ref. [24]; 22 in Ref. [25]; 22 in Ref. [26]).

There is now convincing evidence that altercentric intrusion cannot be accounted for by unspecific attentional and visuo-spatial bias (see Ref. [42]). In contrast with most studies of implicit perspective taking, Santiesteban *et al*. [49] proposed that the mere presence of an avatar gazing to one side of a virtual room redirects spatial attention to this side of the room, thereby accounting for the altercentric intrusion effect. For these authors, altercentric intrusion reflects automatic attentional orienting rather than perspective taking. Because of time constraints in Experiment 1 and the effect of the order of task presentation (see Methods), we could not add another control task presenting an arrow instead of an avatar. Yet, some evidence suggests that when the avatar is replaced by an arrow pointing to one side of the virtual room (which also draws the participant’s attention to this direction), the incongruence of the viewpoint is weaker than when an avatar is presented [25,50]. These data indicate that the presence of the avatar does more than merely draw the participant’s attention to one side of the virtual room.

#### Implicit non-visual perspective taking (graphaesthesia task)

Our results showed that participants implicitly used different perspectives when letters were drawn on their forehead or the back of their head. In many trials (58%), participants used a first-person perspective when ambiguous letters were traced on the forehead but mainly an external, third-person perspective when traced on their back. In addition, 63% of the patients and 63% of the controls preferentially used a first-person perspective to interpret letters drawn on their forehead. This percentage dropped to only 4% for patients and 0% for controls when letters were drawn on the back of their neck. Such percentages are congruent with data from Natsoulas and Dubanoski [27], showing that 70% of the participants preferentially used a first-person perspective for letters drawn on their forehead, whereas 13% used this strategy for letters drawn on the back of their head. Overall, our results agree with previous studies for letters drawn manually by an experimenter [23,27] or automatically with a mechanical device [58]. We note that the fact that an experimenter, instead of a mechanical device drawing letters on the participant’s skin may have increased the likelihood that participants used a third-person perspective. This proposition agrees with implicit perspective taking when a conspecific is located in the participant’s immediate visual environment [24,37].

Another finding of our study was a main effect of the Gender, in that female participants more often used a first-person perspective than did males, which shows an overall stronger anchoring of the self to their body. Gender effects in perspective-taking tasks are controversial, but we have some evidence that females simulate another person’s visuo-spatial perspective [76,77] or perform own-body mental transformation tasks [78] differently from males. In particular, females had longer response times during perspective-taking tasks and were more prone to conflicts between their own body posture and that of a seen individual [76]. Such effects may relate to different cognitive strategies and brain mechanisms used by females and males for mental imagery of objects and bodies, as suggested by early functional neuroimaging studies [79,80].

#### Subjective reports

The IOS scale measuring the perceived closeness between the self and the body did not reveal differences between BVF patients and controls. This result seems to contrast with the greater occurrence of depersonalization-derealization symptoms in vestibular patients than healthy volunteers [64,65,67]. Jauregui-Renaud *et al*. [65] found greater depersonalization-derealization scores for BVF patients than unilateral vestibular-defective patients. Yet, previous studies used a global score of depersonalization-derealization derived from questionnaire items assessing various aspects of the patient’s perception [63]. As a result, whether responses to questionnaire items specifically investigating the anchoring of the self to the body differ for BVF patients and controls remain unknown.

### Limits of the study and future directions

The present findings must be considered with caution because many factors can influence perspective taking and the sample size was limited. Although we controlled for age, gender and education level, which all influence perspective taking [81,76,78], cultural factors [77], personality traits [25,53,78] or anxiety [82] can also play a significant role and may have introduced variability in the data. In addition, we did not perform a power analysis before we included participants; we were constrained by the number of patients with severe BVF, which is a rare condition. Yet, a power analysis for repeated-measures ANOVAs ran *a posteriori* showed that the sample size was not underestimated for Experiments 1 and 2 (G*Power [83]: f = 0.3, α = 0.05, power = 0.8). By contrast, the number of participants was underestimated for Experiment 3, for which a sample size of *n* = 27 per group (instead of *n* = 22) was required (based on a power analysis for Mann-Whitney tests using G*Power [83]: d = 0.8; α = 0.05, power = 0.8).

As noted above, embodiment may be distorted in BVF patients tested with paradigms designed to evoke ‘out-of-the body’ self-locations [9,10,73] and this should be the topic of future investigations. It might also be interesting to evaluate the consequence of acute unilateral vestibular failure (UVF) on anchoring the self to the body. This would allow to compare the consequence of left vs. right UVF as there is an ipsilateral dominance of the vestibulo-thalamo-cortical pathways, and an overall right hemisphere dominance for vestibular information processing in right-handed participants [84,85]. Left and right UVF impact differently visuo-spatial tasks, with a stronger impact of left UVF on the perceived straight-ahead [86], and a stronger impact of right UVF on visual vertical perception [87]. Interestingly, out-of-body experiences have been related to the right temporo-parietal junction [7,11], an important region of the cortical vestibular network [88,89]. Due to the ipsilateral predominance of the vestibulo-thalamo-cortical pathways, patients with right UVF may be more prone to disembodied self-location. This hypothesis should be tested using implicit perspective tasks, such as those used in the present study, and using multisensory conflicts designed to evoke out-of-body—like experiences [9,10,73].

## References

[1] Merleau-Ponty M. Phenomenology of perception. Routledge; 2012.

[2] Claparède E. Note sur la localisation du moi. Arch Psychol. 1925;19: 172–182.

[3] Starmans C, Bloom P. Windows to the soul: Children and adults see the eyes as the location of the self. Cognition. 2012;123: 313–318. 10.1016/j.cognition.2012.02.002 22382132

[4] Blanke O, Dieguez S. Leaving body and life behind: Out-of-body and near-death experience In: Laureys S, Tononi G, editors. The neurology of consciousness: Cognitive neuroscience and neuropathology. Amsterdam: Elsevier; 2009 pp. 303–325.

[5] Blanke O. Multisensory brain mechanisms of bodily self-consciousness. Nat Rev Neurosci. 2012;13: 556–571. 10.1038/nrn3292 22805909

[6] Blanke O, Mohr C. Out-of-body experience, heautoscopy, and autoscopic hallucination of neurological origin. Implications for neurocognitive mechanisms of corporeal awareness and self-consciousness. Brain Res Brain Res Rev. 2005;50: 184–99. 10.1016/j.brainresrev.2005.05.008 16019077

[7] Blanke O, Landis T, Spinelli L, Seeck M. Out-of-body experience and autoscopy of neurological origin. Brain. 2004;127: 243–58. 10.1093/brain/awh040 14662516

[8] Heydrich L, Blanke O. Distinct illusory own-body perceptions caused by damage to posterior insula and extrastriate cortex. Brain. 2013;136: 790–803. 10.1093/brain/aws364 23423672

[9] Lenggenhager B, Tadi T, Metzinger T, Blanke O. Video ergo sum: manipulating bodily self-consciousness. Science. 2007;317: 1096–9. 10.1126/science.1143439 17717189

[10] Ehrsson HH. The experimental induction of out-of-body experiences. Science. 2007;317: 1048 10.1126/science.1142175 17717177

[11] Ionta S, Heydrich L, Lenggenhager B, Mouthon M, Fornari E, Chapuis D, et al Multisensory mechanisms in temporo-parietal cortex support self-location and first-person perspective. Neuron. 2011;70: 363–74. 10.1016/j.neuron.2011.03.009 21521620

[12] Pfeiffer C, Lopez C, Schmutz V, Duenas JA, Martuzzi R, Blanke O. Multisensory origin of the subjective first-person perspective: visual, tactile, and vestibular mechanisms. PloS One. 2013;8: e61751 10.1371/journal.pone.0061751 23630611 PMC3632612

[13] Lenggenhager B, Mouthon M, Blanke O. Spatial aspects of bodily self-consciousness. Conscious Cogn. 2009;18: 110–7. 10.1016/j.concog.2008.11.003 19109039

[14] Blanke O, Pozeg P, Hara M, Heydrich L, Serino A, Yamamoto A, et al Neurological and robot-controlled induction of an apparition. Curr Biol. 2014;24: 2681–2686. 10.1016/j.cub.2014.09.049 25447995

[15] Lenggenhager B, Lopez C. Vestibular contributions to the sense of body, self, and others In: Metzinger T, Windt JM, editors. Open MIND. Frankfurt am Main: MIND-Group; 2015 pp. 1–38.

[16] Lopez C, Halje P, Blanke O. Body ownership and embodiment: Vestibular and multisensory mechanisms. Neurophysiol Clin. 2008;38: 149–61. 10.1016/j.neucli.2007.12.006 18539248

[17] Lopez C. The vestibular system: balancing more than just the body. Curr Opin Neurol. 2016;29: 74–83. 10.1097/WCO.0000000000000286 26679566

[18] Bonnier P. L’Aschématie. Rev Neurol Paris. 1905;12: 605–609. 10.1016/j.yebeh.2009.09.001 19854110

[19] Skworzoff K. Doppelgänger-Halluzinationen bei Kranken mit Funktionsstörungen des Labyrinths. Z Für Gesamte Neurol Psychiatr. 1931;133: 762–766.

[20] Schilder P. The image and appearance of the human body. New York: International Universities Press; 1935.

[21] Grigsby JP, Johnston CL. Depersonalization, vertigo and Meniere’s disease. Psychol Rep. 1989;64: 527–34. 10.2466/pr0.1989.64.2.527 2785275

[22] Lopez C. A neuroscientific account of how vestibular disorders impair bodily self-consciousness. Front Integr Neurosci. 2013;7: 91 10.3389/fnint.2013.00091 24367303 PMC3853866

[23] Ferrè ER, Lopez C, Haggard P. Anchoring the self to the body: vestibular contribution to the sense of self. Psychol Sci. 2014;25: 2106–2108. 10.1177/0956797614547917 25210013

[24] Samson D, Apperly IA, Braithwaite JJ, Andrews BJ, Bodley Scott SE. Seeing it their way: evidence for rapid and involuntary computation of what other people see. J Exp Psychol Hum Percept Perform. 2010;36: 1255–1266. 10.1037/a0018729 20731512

[25] Nielsen MK, Slade L, Levy JP, Holmes A. Inclined to see it your way: Do altercentric intrusion effects in visual perspective taking reflect an intrinsically social process? Q J Exp Psychol 2006. 2015; 1–21. 10.1080/17470218.2015.1023206 25849956

[26] Capozzi F, Cavallo A, Furlanetto T, Becchio C. Altercentric intrusions from multiple perspectives: beyond dyads. PloS One. 2014;9: e114210 10.1371/journal.pone.0114210 25436911 PMC4250177

[27] Natsoulas T, Dubanoski RA. Inferring the locus and orientation of the perceiver from responses to stimulation of the skin. Am J Psychol. 1964;77: 281 14141483

[28] Aron A, Aron EN, Smollan D. Inclusion of Other in the Self Scale and the structure of interpersonal closeness. J Soc Psychol. 1992;63: 596–612.

[29] Demougeot L, Toupet M, Van Nechel C, Papaxanthis C. Action representation in patients with bilateral vestibular impairments. PloS One. 2011;6: e26764 10.1371/journal.pone.0026764 22039548 PMC3200350

[30] Bessot N, Denise P, Toupet M, Van Nechel C, Chavoix C. Interference between walking and a cognitive task is increased in patients with bilateral vestibular loss. Gait Posture. 2012;36: 319–321. 10.1016/j.gaitpost.2012.02.021 22465706

[31] Kapoula Z, Gaertner C, Yang Q, Denise P, Toupet M. Vergence and standing balance in subjects with idiopathic bilateral loss of vestibular function. PloS One. 2013;8: e66652 10.1371/journal.pone.0066652 23825551 PMC3688965

[32] MacDougall HG, Weber KP, McGarvie LA, Halmagyi GM, Curthoys IS. The video head impulse test Diagnostic accuracy in peripheral vestibulopathy. Neurology. 2009;73: 1134–1141. 10.1212/WNL.0b013e3181bacf85 19805730 PMC2890997

[33] Welgampola MS, Colebatch JG. Characteristics and clinical applications of vestibular-evoked myogenic potentials. Neurology. 2005;64: 1682–8. 10.1212/01.WNL.0000161876.20552.AA 15911791

[34] Rosengren SM, McAngus Todd NP, Colebatch JG. Vestibular-evoked extraocular potentials produced by stimulation with bone-conducted sound. Clin Neurophysiol. 2005;116: 1938–48. 10.1016/j.clinph.2005.03.019 15979939

[35] Baloh RW, Honrubia V. Clinical Neurophysiology of the Vestibular System. Oxford University Press; 2001. 378525

[36] McGarvie LA, MacDougall HG, Halmagyi GM, Burgess AM, Weber KP, Curthoys IS. The video head impulse test (vHIT) of semicircular canal function—age-dependent normative values of VOR gain in healthy subjects. Front Neurol. 2015;6: 154 10.3389/fneur.2015.00154 26217301 PMC4495346

[37] Tversky B, Hard BM. Embodied and disembodied cognition: spatial perspective-taking. Cognition. 2009;110: 124–129. 10.1016/j.cognition.2008.10.008 19056081

[38] Mazzarella E, Hamilton A, Trojano L, Mastromauro B, Conson M. Observation of another’s action but not eye gaze triggers allocentric visual perspective. Q J Exp Psychol 2006. 2012;65: 2447–2460. 10.1080/17470218.2012.697905 22901326

[39] Furlanetto T, Cavallo A, Manera V, Tversky B, Becchio C. Through your eyes: incongruence of gaze and action increases spontaneous perspective taking. Front Hum Neurosci. 2013;7: 455 10.3389/fnhum.2013.00455 23964228 PMC3740297

[40] Surtees ADR, Apperly IA. Egocentrism and automatic perspective taking in children and adults. Child Dev. 2012;83: 452–460. 10.1111/j.1467-8624.2011.01730.x 22335247

[41] Ramsey R, Hansen P, Apperly I, Samson D. Seeing it my way or your way: frontoparietal brain areas sustain viewpoint-independent perspective selection processes. J Cogn Neurosci. 2013;25: 670–684. 10.1162/jocn_a_00345 23249349

[42] Furlanetto T, Becchio C, Samson D, Apperly I. Altercentric interference in level 1 visual perspective taking reflects the ascription of mental states, not submentalizing. J Exp Psychol Hum Percept Perform. 2016;42: 158–163. 10.1037/xhp0000138 26389611

[43] Mattan B, Quinn KA, Apperly IA, Sui J, Rotshtein P. Is It Always Me First? Effects of Self-Tagging on Third-Person Perspective-Taking. J Exp Psychol Learn Mem Cogn. 2014 10.1037/xlm0000078 25528086

[44] Qureshi AW, Apperly IA, Samson D. Executive function is necessary for perspective selection, not Level-1 visual perspective calculation: evidence from a dual-task study of adults. Cognition. 2010;117: 230–236. 10.1016/j.cognition.2010.08.003 20817158

[45] Deroualle D, Borel L, Devèze A, Lopez C. Changing perspective: The role of vestibular signals. Neuropsychologia. 2015;79, Part B: 175–185. 26311354 10.1016/j.neuropsychologia.2015.08.022

[46] Deroualle D, Hautefort C, Van Nechel C, Duquesne U, Toupet M, Lopez C. Une perte vestibulaire bilatérale modifie-t-elle l’ancrage du soi sur le corps dans des tâches de prise de perspective? Neurophysiol Clin Neurophysiol. 2015;45: 400–401.

[47] Oldfield RC. The assessment and analysis of handedness: the Edinburgh inventory. Neuropsychologia. 1971;9: 97–113. 5146491 10.1016/0028-3932(71)90067-4

[48] Peirce JW. PsychoPy—Psychophysics software in Python. J Neurosci Methods. 2007;162: 8–13. 10.1016/j.jneumeth.2006.11.017 17254636 PMC2018741

[49] Santiesteban I, Catmur C, Hopkins SC, Bird G, Heyes C. Avatars and arrows: implicit mentalizing or domain-general processing? J Exp Psychol Hum Percept Perform. 2014;40: 929–937. 10.1037/a0035175 24377486

[50] Lopez C, Parlanti A, Deroualle D. La posture influence de façon préconsciente la perception visuo-spatiale. Neurophysiol Clin Neurophysiol. 2014;44: 494–495.

[51] Heed T, Habets B, Sebanz N, Knoblich G. Others’ actions reduce crossmodal integration in peripersonal space. Curr Biol. 2010;20: 1345–9. 10.1016/j.cub.2010.05.068 20619649

[52] Aspell JE, Lenggenhager B, Blanke O. Keeping in touch with one’s self: multisensory mechanisms of self-consciousness. PLoS One. 2009;4: e6488 10.1371/journal.pone.0006488 19654862 PMC2715165

[53] Lopez C, Falconer CJ, Mast FW. Being moved by the self and others: influence of empathy on self-motion perception. PloS One. 2013;8: e48293 10.1371/journal.pone.0048293 23326302 PMC3543431

[54] Grabherr L, Cuffel C, Guyot JP, Mast FW. Mental transformation abilities in patients with unilateral and bilateral vestibular loss. Exp Brain Res. 2011;209: 205–14. 10.1007/s00221-011-2535-0 21287158

[55] Candidi M, Micarelli A, Viziano A, Aglioti SM, Minio-Paluello I, Alessandrini M. Impaired mental rotation in benign paroxysmal positional vertigo and acute vestibular neuritis. Front Hum Neurosci. 2013;7: 783 10.3389/fnhum.2013.00783 24324422 PMC3840898

[56] Péruch P, Lopez C, Redon C, Escoffier G, Zeitoun A, Sanjuane M, et al Vestibular information is necessary for maintaining metric properties of representational space: Evidence from mental imagery. Neuropsychologia. 2011;49: 3136–3144. 10.1016/j.neuropsychologia.2011.07.026 21820000

[57] Natsoulas T. Locus and orientation of the perceiver (ego) under variable, constant, and no perspective instructions. J Pers Soc Psychol. 1966;3: 190–196. 5903527 10.1037/h0022815

[58] Arnold G, Spence C, Auvray M. Taking someone else’s spatial perspective: Natural stance or effortful decentring? Cognition. 2016;148: 27–33. 10.1016/j.cognition.2015.12.006 26722709

[59] Spence C, Ngo MK, Lee J-H, Tan H. Solving the correspondence problem in haptic/multisensory interface design In: Zadeh MH, editor. Advances in haptics. InTech; 2010 pp. 47–74. Available: http://www.intechopen.com/books/advances-in-haptics/solving-thecorrespondence-problem-in-haptic-multisensory-interface-design

[60] Corcoran DW. The phenomena of the disembodied eye or is it a matter of personal geography? Perception. 1977;6: 247–253. 866080 10.1068/p060247

[61] Parsons LM, Shimojo S. Perceived spatial organization of cutaneous patterns on surfaces of the human body in various positions. J Exp Psychol Hum Percept Perform. 1987;13: 488–504. 2958596 10.1037//0096-1523.13.3.488

[62] Sekiyama K. Importance of head axes in perception of cutaneous patterns drawn on vertical body surfaces. Percept Psychophys. 1991;49: 481–492. 2057314 10.3758/bf03212182

[63] Cox BJ, Swinson RP. Instrument to assess depersonalization-derealization in panic disorder. Depress Anxiety. 2002;15: 172–5. 10.1002/da.10051 12112722

[64] Sang FY, Jauregui-Renaud K, Green DA, Bronstein AM, Gresty MA. Depersonalisation/derealisation symptoms in vestibular disease. J Neurol Neurosurg Psychiatry. 2006;77: 760–6. 10.1136/jnnp.2005.075473 16464901 PMC2077438

[65] Jauregui-Renaud K, Sang FY, Gresty MA, Green DA, Bronstein AM. Depersonalisation/derealisation symptoms and updating orientation in patients with vestibular disease. J Neurol Neurosurg Psychiatry. 2008;79: 276–83. 10.1136/jnnp.2007.122119 17578858

[66] Jauregui-Renaud K, Ramos-Toledo V, Aguilar-Bolanos M, Montano-Velazquez B, Pliego-Maldonado A. Symptoms of detachment from the self or from the environment in patients with an acquired deficiency of the special senses. J Vestib Res. 2008;18: 129–37. 19126983

[67] Kolev OI, Georgieva-Zhostova SO, Berthoz A. Anxiety changes depersonalization and derealization symptoms in vestibular patients. Behav Neurol. 2014;2014: 847054 10.1155/2014/847054 24803735 PMC4006595

[68] Kaliuzhna M, Vibert D, Grivaz P, Blanke O. Out-of-body experiences and other complex dissociation experiences in a patient with unilateral peripheral vestibular damage and deficient multisensory integration. Multisensory Res. 2015;28: 613–635. 10.1163/22134808-00002506 26595959

[69] Fanelli D. Do pressures to publish increase scientists’ bias? An empirical support from US states data. PLoS ONE. 2010;5. 10.1371/journal.pone.0010271 PMC2858206 20422014

[70] Matosin N, Frank E, Engel M, Lum JS, Newell KA. Negativity towards negative results: a discussion of the disconnect between scientific worth and scientific culture. Dis Model Mech. 2014;7: 171–173. 10.1242/dmm.015123 24713271 PMC3917235

[71] Heydrich L, Lopez C, Seeck M, Blanke O. Partial and full own-body illusions of epileptic origin in a child with right temporoparietal epilepsy. Epilepsy Behav. 2011;20: 583–6. 10.1016/j.yebeh.2011.01.008 21334265

[72] Guterstam A, Björnsdotter M, Gentile G, Ehrsson HH. Posterior cingulate cortex integrates the senses of self-location and body ownership. Curr Biol. 2015;25: 1416–1425. 10.1016/j.cub.2015.03.059 25936550

[73] Guterstam A, Ehrsson HH. Disowning one’s seen real body during an out-of-body illusion. Conscious Cogn. 2012;21: 1037–1042. 10.1016/j.concog.2012.01.018 22377139

[74] Dieguez S, Lopez C. The bodily self: Insights from clinical and experimental research. Ann Phys Rehabil Med. 10.1016/j.rehab.2016.04.007 27318928

[75] Bronstein AM, Yardley L, Moore AP, Cleeves L. Visually and posturally mediated tilt illusion in Parkinson’s disease and in labyrinthine defective subjects. Neurology. 1996;47: 651–6. 8797458 10.1212/wnl.47.3.651

[76] Kessler K, Wang H. Spatial perspective taking is an embodied process, but not for everyone in the same way: differences predicted by sex and social skills score. Spat Cogn Comput. 2012;12: 133–158.

[77] Kessler K, Cao L, O’Shea KJ, Wang H. A cross-culture, cross-gender comparison of perspective taking mechanisms. Proc Biol Sci. 2014;281: 20140388 10.1098/rspb.2014.0388 24807256 PMC4024296

[78] Mohr C, Rowe AC, Blanke O. The influence of sex and empathy on putting oneself in the shoes of others. Br J Psychol. 2010;101: 277–291. 10.1348/000712609X457450 19619391

[79] Butler T, Imperato-McGinley J, Pan H, Voyer D, Cordero J, Zhu YS, et al Sex differences in mental rotation: top-down versus bottom-up processing. Neuroimage. 2006;32: 445–56. 10.1016/j.neuroimage.2006.03.030 16714123

[80] Hugdahl K, Thomsen T, Ersland L. Sex differences in visuo-spatial processing: an fMRI study of mental rotation. Neuropsychologia. 2006;44: 1575–83. 10.1016/j.neuropsychologia.2006.01.026 16678867

[81] Mattan BD, Quinn KA, Acaster SL, Jennings RM, Rotshtein P. Prioritization of self-relevant perspectives in ageing. Q J Exp Psychol 2006. 2016; 1–20. 10.1080/17470218.2015.1127399 26652616

[82] Todd AR, Forstmann M, Burgmer P, Brooks AW, Galinsky AD. Anxious and egocentric: how specific emotions influence perspective taking. J Exp Psychol Gen. 2015;144: 374–391. 10.1037/xge0000048 25602753

[83] Faul F, Erdfelder E, Lang A-G, Buchner A. G*Power 3: A flexible statistical power analysis program for the social, behavioral, and biomedical sciences. Behav Res Methods. 2007;39: 175–191. 17695343 10.3758/bf03193146

[84] Dieterich M, Bense S, Lutz S, Drzezga A, Stephan T, Bartenstein P, et al Dominance for vestibular cortical function in the non-dominant hemisphere. Cereb Cortex. 2003;13: 994–1007. 12902399 10.1093/cercor/13.9.994

[85] Janzen J, Schlindwein P, Bense S, Bauermann T, Vucurevic G, Stoeter P, et al Neural correlates of hemispheric dominance and ipsilaterality within the vestibular system. Neuroimage. 2008;42: 1508–18. 10.1016/j.neuroimage.2008.06.026 18644454

[86] Saj A, Honoré J, Bernard-Demanze L, Devèze A, Magnan J, Borel L. Where is straight ahead to a patient with unilateral vestibular loss? Cortex. 2013;49: 1219–1228. 10.1016/j.cortex.2012.05.019 22795184

[87] Toupet M, Van Nechel C, Bozorg Grayeli A. Influence of body laterality on recovery from subjective visual vertical tilt after vestibular neuritis. Audiol Neurootol. 2014;19: 248–255. 10.1159/000360266 25074802

[88] Kahane P, Hoffmann D, Minotti L, Berthoz A. Reappraisal of the human vestibular cortex by cortical electrical stimulation study. Ann Neurol. 2003;54: 615–24. 10.1002/ana.10726 14595651

[89] Lopez C, Blanke O. The thalamocortical vestibular system in animals and humans. Brain Res Rev. 2011;67: 119–146. 10.1016/j.brainresrev.2010.12.002 21223979

